# Novel application of multi-loop traction device and threaded clip in gastric endoscopic submucosal dissection

**DOI:** 10.1055/a-2623-4478

**Published:** 2025-06-17

**Authors:** Hossam El-Din Shaaban, Keisaku Yamada, Masahiro Tajika, Tsutomu Tanaka, Nobuhito Ito, Akihiro Takagi, Yasumasa Niwa

**Affiliations:** 1 538357 Endoscopy, Aichi Cancer Center, Nagoya, Japan

Traction-assisted endoscopic submucosal dissection (ESD) employing a dental floss clip has demonstrated usefulness in gastric ESD 1 . However, it provides insufficient traction for large lesions when a single traction point is used. We developed a novel traction technique using a Multi-Loop Traction Device (MLTD; Boston Scientific Co. Ltd., Tokyo, Japan) that enables traction on three points with a single traction. We named this the “anchor traction method” 2 . Here we report application of this technique on a large lesion by combining MLTD and a threaded clip to achieve effective traction at three points.

**Fig. 1:**
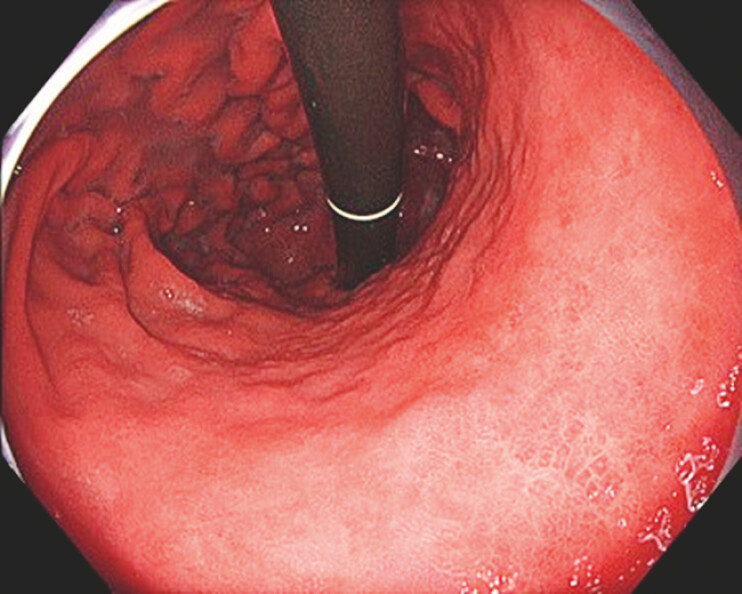
The lesion was a 55-mm 0-IIc lesion on the lesser curvature of the gastric body.

A 68-year-old male presented with a 55-mm IIc lesion on the lesser curvature of the gastric body ( Fig. 1 ). He then underwent ESD ( Video 1 ).

Novel application of MLTD and a threaded clip in gastric ESD.Video 1

**Fig. 2:**
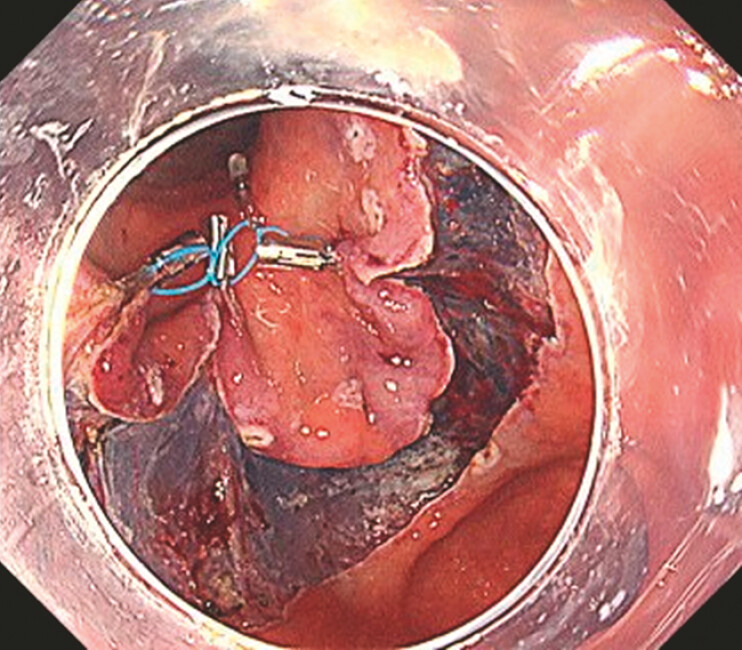
The combination of MLTD with a threaded clip can provide effective traction.

**Fig. 3:**
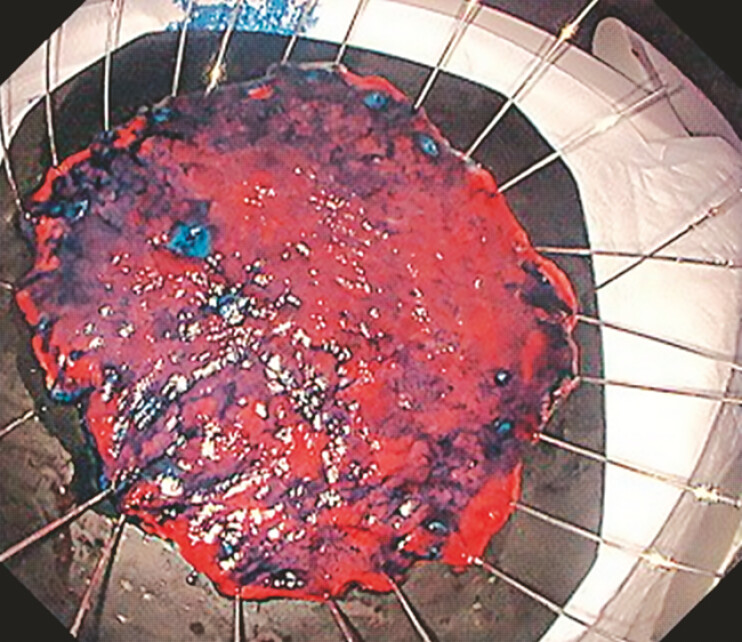
The histopathology result was 0-IIc, 55 × 30 mm, tub2 > por1, pT1b (SM, 900 µm), INFa, LY0, V0, UL0, pHM0, pVM0.

A full circumferential incision was made and the middle loop of the MLTD was grasped with a reopenable clip (SureClip; MicroTech, Nanjing, China) and attached to the anal side of the lesion. Two additional loops of MLTD were then attached to the lesion, employing the “anchor traction method”. The scope was removed and a clip with thread was attached, then the scope was reinserted. The clip was then attached to the middle loop of the MLTD. The thread was pulled to obtain good traction ( Fig. 2 ). Traction on multiple points enabled safe ESD using the IT knife 2. Histopathology result was 0-IIc, 55×30mm, tub2 > por1, pT1b (SM, 900 µm), INFa, LY0, V0, UL0, pHM0, pVM0 ( Fig. 3 ), and the patient underwent additional surgery later.

Although there are already reports of traction devices allowing traction in multiple locations 3 4 , when treating lesions such as these in retroflex position, the traction device may interfere with the scope, causing traction to be removed. Therefore, the combination of MLTD with a threaded clip can provide effective traction in gastric ESD.
